# Anthocyanins do not influence long-chain n-3 fatty acid status: studies in cells, rodents and humans^☆^

**DOI:** 10.1016/j.jnutbio.2014.09.005

**Published:** 2015-03

**Authors:** David Vauzour, Noemi Tejera, Colette O'Neill, Valeria Booz, Baptiste Jude, Insa M.A. Wolf, Neil Rigby, Jose Manuel Silvan, Peter J. Curtis, Aedin Cassidy, Sonia de Pascual-Teresa, Gerald Rimbach, Anne Marie Minihane

**Affiliations:** a Department of Nutrition, Norwich Medical School, Faculty of Medicine and Health Sciences, University of East Anglia, Norwich, NR4 7TJ, United Kingdom; b Institute of Food Research, Norwich Research Park, Norwich NR4 7UA, United Kingdom; c Deparment of Metabolism and Nutrition, Instituto de Ciencia y Tecnología de Alimentos y Nutrición (ICTAN), CSIC, José Antonio Novais 10, 28040 Madrid, Spain; d Institute of Human Nutrition and Food Science, Christian-Albrechts-University, D-24118 Kiel, Germany

**Keywords:** Anthocyanins, n-3 PUFA, Liver, Rat, Human

## Abstract

Increased tissue status of the long-chain n-3 polyunsaturated fatty acids (LC n-3 PUFA), eicosapentaenoic (EPA) and docosahexaenoic acid (DHA) is associated with cardiovascular and cognitive benefits. Limited epidemiological and animal data suggest that flavonoids, and specifically anthocyanins, may increase EPA and DHA levels, potentially by increasing their synthesis from the shorter-chain n-3 PUFA, α-linolenic acid. Using complimentary cell, rodent and human studies we investigated the impact of anthocyanins and anthocyanin-rich foods/extracts on plasma and tissue EPA and DHA levels and on the expression of fatty acid desaturase 2 (*FADS2*), which represents the rate limiting enzymes in EPA and DHA synthesis. In experiment 1, rats were fed a standard diet containing either palm oil or rapeseed oil supplemented with pure anthocyanins for 8 weeks. Retrospective fatty acid analysis was conducted on plasma samples collected from a human randomized controlled trial where participants consumed an elderberry extract for 12 weeks (experiment 2). HepG2 cells were cultured with α-linolenic acid with or without select anthocyanins and their *in vivo* metabolites for 24 h and 48 h (experiment 3). The fatty acid composition of the cell membranes, plasma and liver tissues were analyzed by gas chromatography. Anthocyanins and anthocyanin-rich food intake had no significant impact on EPA or DHA status or *FADS2* gene expression in any model system. These data indicate little impact of dietary anthocyanins on n-3 PUFA distribution and suggest that the increasingly recognized benefits of anthocyanins are unlikely to be the result of a beneficial impact on tissue fatty acid status.

## 1 Introduction

Although not fully consistent, there are substantial data to indicate that increased dietary intake and tissue status of the long-chain n-3 polyunsaturated fatty acid (LC n-3 PUFA), eicosapentaenoic acid (C20:5-n3; EPA) and docosahexaenoic acid (C22:6n3; DHA) are associated with cardiovascular and cognitive benefits [1–3]. While a minimum intake of 0.5 g EPA+DHA per day is currently typically recommended for adults [4,5], population-based estimates suggest the average combined intake of EPA and DHA is <0.2 g per day, with <0.05 g per day consumed by a large proportion of adult populations, i.e., >10-fold lower than the minimum recommended intake [6,7]. Accessibility, affordability and palatability of fish products are often cited as reasons for low EPA and DHA intakes [8]. Also, sustainability of fish stocks is of major concern, with the current production of 1 million tons of fish oils per year insufficient to meet recommended intakes. Therefore alternative strategies to increase EPA+DHA status are needed.

**Fig. 1:**
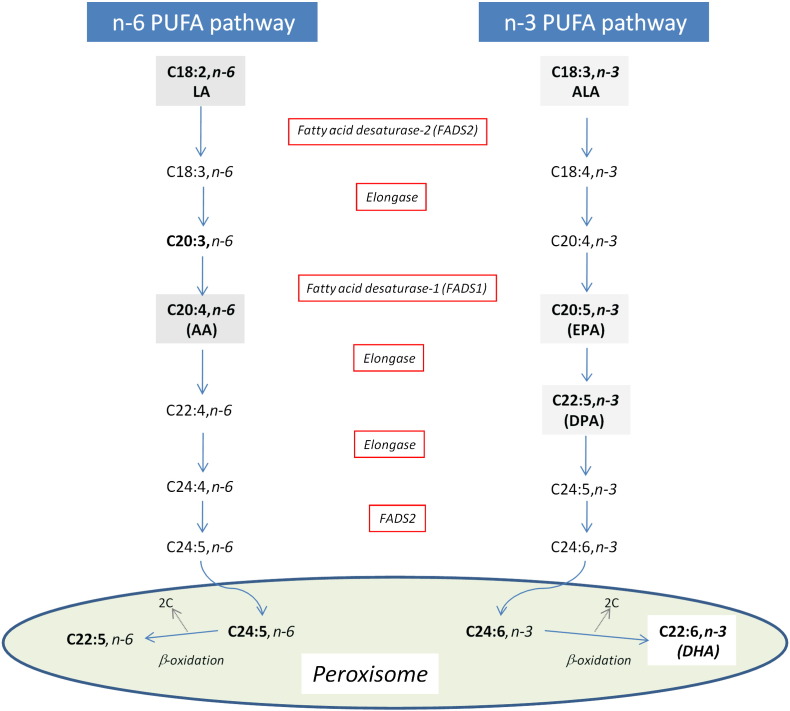
Synthesis of long-chain PUFA from linoleic (LA) acid and ALA (n-3). Both LA (n-6) and ALA (n-3) are elongated, desaturated and β-oxidized using the same enzyme system. AA, arachidonic acid.

In mammals, EPA and, to a lesser extent, DHA can be synthesized from the shorter-chain plant derived n-3 PUFA, α-linolenic acid (C18:3n-3; ALA) (Fig. 1), with the biosynthesis occurring predominantly in the liver. With the exception of premenopausal women, in whom the conversion of 18:3n-3 to 20:5n-3 and 22:6n-3 is substantially greater, the bioconversion of ALA is usually inefficient and does not exceed 0.2%–6% for EPA and 0.1% for DHA [9]. Limited evidence is suggestive that plant-derived bioactives may increase the conversion efficiency. Vegetarians, and vegans have adequate status despite negligible intake [7]. In observational studies, alcoholic beverages and specifically wine consumption [rich in anthocyanins (ACNs)] have been associated with increased LC n-3 PUFA status [10–13]. In a feeding study in rats, the inclusion of ACNs (found in red grapes, red wine and berry fruits) in the diet was associated with 20%–35% and 10%–20% increased plasma EPA and DHA [14] with the authors suggesting that increased bioconversion of ALA to EPA+DHA may be partly responsible (Fig. 1). More recently, rats fed with ACN-rich grape–bilberry juice for 10 weeks had an increased overall percentage of PUFAs and a decreased percentage saturated fatty acids (SFAs) in plasma. However, no signification impact on LC n-3 PUFA concentrations were observed [15].

Using rodent studies, we investigated the impact of flavonoids/ACNs on EPA and DHA status in plasma, and for the first time in liver tissue, with hepatic gene expression of fatty acid desaturase 2 (*FADS2*), the rate-limiting enzyme in the ALA to EPA and DHA bioconversion pathway (Fig. 1) also investigated. Stored samples from a previously completed randomized controlled trial [16] in our laboratory were analyzed to establish the impact of an ACN-rich food extract on LC n-3 PUFA status in humans. Finally, cell culture studies in HepG2 cells were used to explore the effect of a range of ACNs and their metabolites on fatty acid status and *FADS2* gene expression.

## 2 Methods and materials

### 2.1 Chemicals and reagents

Bovine serum albumin (BSA; fatty acid free), ALA, DHA, EPA, nonadecanoic acid (19:0), tridecanoic acid, gallic acid (GA), *p*-coumaric acid (PCA), syringic acid (SYA), butylated hydroxytoluene (BHT), potassium chloride and potassium bicarbonate were obtained from Sigma-Aldrich. Thin layer chromatography (TLC) (20×20 cm) plates, precoated with silica gel (without fluorescent indicator) were purchased from Macherey-Nagel (Düren, Germany). A fish oil standard (PUFA n-3) was supplied by Supelco (Supelco Park, Bellefonte, PA, USA). Delphinidin-3-*O*-glucoside (D3G), cyanidin-3-*O*-glucoside (C3G) and malvidin-3-*O*-glucoside (M3G) were obtained from Extrasynthese (Genay, France). Palm oil (PO) and rapeseed oil (RO) were obtained from William Hodgson & Co (Congleton, UK). All HPLC-grade solvents for chromatography and solvents for extraction (proanalysis quality) were purchased from Fisher Scientific (Fisher Scientific UK Ltd). The test polyphenolic compounds were dissolved in DMSO (Carl Roth, Karlsruhe, Germany), and stock solutions were stored at −80°C until usage.

### 2.2 Animals and diets

All experimental procedures and protocols used in this study were reviewed and approved by the Animal Welfare and Ethical Review Body and were conducted according to the specifications of the United Kingdom Animals (Scientific Procedures) Act, 1986.

Male Wistar rats aged 7–8 weeks were purchased from Charles River Laboratories (Margate, UK). Rats were housed two per cage and under conditions of constant temperature (21±2°C), humidity (55%±10%) and a standard light–dark cycle (12 h/12 h) and were fed a standard RM3 diet (Special Diet Services, UK) while acclimatizing. Rats were given food and water *ad libitum*. The test diets were prepared by replacing the fat content by PO or RO into the standard purified diet for rodents D12450B (Research Diets Inc, New Brunswick, NJ, USA). PO was chosen as the control oil as it contains only trace amounts of ALA and has a high SFA content typical of a Westernized-type diet. RO is a commonly consumed rich source of ALA which typically constitutes around 10% of its total fatty acids. The nutrient composition of the experimental diets and the fatty acid composition of the dietary fats are given in Supplementary Tables 1 and 2, respectively.

Male Wistar rats aged 7–8 weeks were purchased from Charles River Laboratories (Margate, UK). Rats were housed two per cage and under conditions of constant temperature (21±2°C), humidity (55%±10%) and a standard light–dark cycle (12 h/12 h) and were fed a standard RM3 diet (Special Diet Services, UK) while acclimatizing. Rats were given food and water *ad libitum*. The test diets were prepared by replacing the fat content by PO or RO into the standard purified diet for rodents D12450B (Research Diets Inc, New Brunswick, NJ, USA). PO was chosen as the control oil as it contains only trace amounts of ALA and has a high SFA content typical of a Westernized-type diet. RO is a commonly consumed rich source of ALA which typically constitutes around 10% of its total fatty acids. The nutrient composition of the experimental diets and the fatty acid composition of the dietary fats are given in Supplementary Tables 1 and 2, respectively.

Rats were randomly distributed into four groups of 10 animals each, in a 2×2 factorial design. Two groups of rats were fed the diet without supplementation (PO and RO), whereas the other two received the same diets supplemented with 240 mg/kg diet of dietary ACNs (PO+ACN and RO+ACN). The ACN fraction was prepared by extracting ACNs from blueberries giving rise to a prepurified extract. HPLC analysis [17,18] indicated that the diet was composed of 38.3% of delphinidin-3-*O*-β-glucopyranoside, 34.7% of malvidin-3-*O*-β-glucopyranoside, 18.8% of petunidin-3-O-β-glucopyranoside, 6.7% of cyanidin-3-*O*-β-glucopyranoside and 1.4% of peonidin 3-*O*-β-glucopyranoside). No ACNs were detected in the RO and PO control diets. Body weight and food intake were recorded three times a week. At the end of the 8-week intervention, rats were anesthetized with an overdose of sodium pentobarbital (150 mg/kg, i.p,) and blood was drawn by cardiac puncture into serum separation tubes (SST, Becton Dickinson, UK). Rats were then transcardially perfused with an ice cold saline solution (100 ml) containing 10 U/ml of sodium heparin. Serum was obtained after centrifugation (10 min, 1300×*g*, room temperature) and immediately frozen at −80°C until analysis. A portion of liver was rapidly removed, rinsed with ice-cold 150 mmol/L NaCl, blotted and weighed. Individual aliquots were snap-frozen and stored at −80°C before extraction of lipids.

### 2.3 Human plasma samples

Stored plasma samples from our completed randomized control trial were used to examine the impact of ACNs on fatty acid status in humans. In this placebo-controlled intervention designed to examine the impact of ACN-rich elderberry extract on biomarkers of cardiovascular health, postmenopausal women consumed 500 mg/day ACNs as cyanidin glycosides (from elderberry) for 12 weeks. The study design has been previously described in detail [16]. The ACN content of the elderberry (*Sambucus nigra*) extract was previously established as 250 mg ACN per gram of extract [16], predominantly consisting of cyanidin-3-glucoside (53.5%) and cyanidin-3-sambubioside (39.5%) [19]. Elderberries are also reported to contain low amounts of cyanidin-3-sambubioside-5-glucoside (6%) and cyanidin-3,5-diglucoside (1%) [20]. Volunteers were on average 8y postmenopausal with the groups matched for age and body mass index (BMI), with a mean age and BMI of 58.3 years and 24.3 kg/m^2^ and 58.1 years and 25.1 kg/m^2^ in the placebo and ACN groups, respectively. At the beginning and end of the intervention, participants provided a 12-h fasting blood sample collected into K2-EDTA. Plasma was obtained by centrifugation at 1500×*g* for 15 min within 30 min of collection and stored at −80°C awaiting analysis.

### 2.4 HepG2 cell culture and treatments

The HepG2 liver cell line (LGC Standards) were maintained in Dulbecco's modified Eagle's medium (DMEM) containing 4.5 g/L glucose, 4 mmol/L l-glutamine, 1 mmol/L sodium pyruvate, 10% heat-inactivated fetal calf serum (FCS), 100 U/ml penicillin and 100 μg/ml streptomycin (PAA, Coelbe, Germany). Cells were grown in 5% CO_2_ at 37°C under a humidified atmosphere. All cell-culture plasticware was purchased from Sarstedt (Nuembrecht, Germany) unless otherwise stated. For the experiments, cells were seeded (2×10^6^ cells/well) into 6-well plates and cultured for 24 h in routine culture medium at 37°C, under 5% CO_2_ and 95% air. The routine culture medium was aspirated and the HepG2 cells were washed twice with phosphate-buffered saline and cultured for 24 h in serum-free DMEM. Serum-free conditions were used in order to avoid potential flavonoid–protein interaction [21] and to minimize interference of other fatty acids present in FCS [22]. Individual aliquots of ALA-BSA (1:2 molar ratio, 50 μM ALA) working solution alone (control) or supplemented with D3G, C3G and M3G and their metabolites, GA, SYA and PCA (5 μM) were added to the HepG2 cell culture. Time points and ALA concentration were chosen according to previously published data [23,24]. ACNs subclasses were chosen as they represent the main dietary ACNs and/or the main ACN metabolites found in the systemic circulation [25]. Concentrations of ALA and ACN metabolites of 50 and 5 μM were chosen as they represent achievable levels in human plasma and therefore a physiologically relevant hepatocyte exposure [26,27]. Assays vehicle controls were included which did not affect any of the parameters measured.

Cytotoxicity of the pure ACNs and their metabolites was determined via the Neutral Red Assay [28]. Briefly, HepG2 cells were seeded in 24-well plates, precultured for 24 h and treated with 25 and 50 μM of the test compounds for 48 h. The culture medium containing the test substances was replaced with fresh serum-containing medium including 50 μg/ml of Neutral Red (Carl Roth). After incubation for 3 h, the medium was removed and the cells were extracted using a solution comprising 50:49:1 (vol/vol/vol) ethanol, water and glacial acetic acid. The absorbance was measured in a plate reader (Labsystems, Helsinki, Finland) at 540 nm.

### 2.5 RNA isolation and real-time PCR

For RNA-isolation, HepG2 cells were precultured in 6-well plates for 24 h. Subsequently, cells were serum starved for 24 h. Then cells were incubated for 48 h with 50 µmol/L of the test compounds in serum free medium supplemented with ALA-BSA. RNA was isolated with TRIfast following the manufacturer's protocol (Peqlab, Hamburg, Germany). FADS2 and GAPDH primers were designed by primer3 software with the following sequences: *HuFADS2*: ACA AGG ATC CCG ATG TGA AC (forward) and TTC GTG CTG GTG ATT GTA GG (reverse); *HuGAPDH*: CAA TGA CCC CTT CAT TGA CC (forward) and GAT CTC GCT CCT GGA AGA TG (reverse) (MWG Biotech, Ebersberg, Germany). Real-time PCR was performed using Sensi-Mix one-step kit (Quantace, Berlin, Germany).

For liver samples, total RNA was extracted with Trizol reagent (Invitrogen Life Technologies, Paisley, UK). *FADS2* and *GAPDH* primers were designed by primer3 software with the following sequences: *rnFADS2*: CCC AAG CTG GAT GGC TAC AA (forward) and TGC AGG CTC TTT ATG TCG GG (reverse); *rnGAPDH*: GTC TAC TGG CGT CTT CAC CA (forward) and GTG GCA GTG ATG GCA TGG AC (reverse) (Life Technology, UK). The reverse transcriptase reaction was performed with 5 μg of total RNA using SuperScript II Reverse Transcriptase (Invitrogen) and oligo (dT) primers. The RT-qPCR analysis was performed using a PCR master mix with SYBR green (PrimerDesign Ltd, Southampton, UK) with an Applied Biosystems 7500 Real time PCR. Results were normalized using GAPDH as a reference. After normalization, all results are expressed as a percentage of control as means and standard error means (S.E.M).

### 2.6 Lipid and fatty acid analysis

The fatty acid composition of the HepG2 cells was determined by GC as described previously [29]. Briefly, after a period of 24 and 48 h, the medium was aspirated and the adherent cells were removed from the plates using a plastic tissue culture plate scraper and centrifuged for 5 min at 2000×*g*. After centrifugation, the harvested cells were resuspended in 200 μL 0.2 M NaCl and cell lipids were extracted using 1 ml chloroform/methanol (2:1, vol/vol). Fatty acid methyl esters (FAMEs) were synthesized by incubation with methanolic sulphuric acid (2% H_2_SO_4_, vol/vol) at 50°C for 2 h followed by extraction with hexane. FAMEs were resolved on a 7820A Agilent gas chromatograph (Agilent) equipped with a 60 m×250 μM×0.2 μM Agilent HP-88 fused silica capillary column using the following temperature protocol: initial temperature 115°C, ramp 8°C/min to 145°C (26 min), ramp 2°C/min to 220°C (5 min). FAMEs were identified and quantified by comparison with ALA, EPA and DHA methyl ester standards run previously. Chromatograms were recorded with an EZChrom Elite data system and results are presented as percentage of total fatty acids.

For human and rodent samples, total lipids were extracted from 500 μl of plasma or 300 mg of liver with chloroform/methanol (2:1 vol/vol) containing 0.01% BHT as antioxidant [30]. The organic solvent was evaporated under a stream of nitrogen and the lipid content was determined gravimetrically. Two milligrams of the total lipid fraction (TL) was subjected to acid-catalyzed transmethylation for 16 h at 50°C, using 1 ml of toluene and 2 ml of 1% sulphuric acid (vol/vol) in methanol. Prior to transmethylation, nonadecanoic acid (19:0) (5% of the total lipid analyzed), was added as internal standard to the lipid extracts. The resultant FAMEs were purified by TLC and visualized under spraying with 1% iodine in chloroform [31]. After elution, FAMEs were separated and quantified by gas–liquid chromatography using a Hewlett Packard 5890 GC and a SGE BPX70 capillary GC column (30 m×0.22 mm I.D.; SGE UK Ltd) with helium as carrier gas and using on-column injection. The temperature gradient started at 115°C for 3 min, then went to 200°C at 2°C/min, 2 min at 200°C, and then to 240°C at 60°C/min. After 5 min at 240°C, it cooled down to 115°C and equilibrated for 3 min before the next injection. Individual methyl esters were identified by reference to authentic standards and to well-characterized fish oil (PUFA-3 from menhaden oil; Supelco). Data were collected and processed using GC Chemstation (version B04-02).

### 2.7 FADS2 activity index

The activity of FADS2 (also termed Δ6-desaturase) was estimated by the ratio 18:3n-3/18:2n-6 in the human samples [32].

### 2.8 Statistical analysis

Results are presented as means±S.D. with significance at *P*<.05. The data were checked for normal distribution with the one-sample Kolmogorov–Smirnoff test as well as for homogeneity of variance with the Levene test and were log-transformed where necessary before statistical analysis. Two-way analysis of variance (ANOVA) was used to test effects of dietary fat type, supplementation with ACNs and their interaction on variables in the rodent study or time, supplementation with ACNs/ACNs-rich food and their interaction on variables in the human trial and the cell work. The differences between means were tested using the Tukey's multiple comparison test when the *F* value was significant [33]. One-way ANOVA was used to test the effects of the type of fat and the ACNs on the growth parameters, liver weight and desaturase mRNA levels. The statistical analysis was performed by using the SPSS package (version 16.0; SPSS Inc., Chicago, IL, USA).

## 3 Results

### 3.1 Rodent study

There was a significant increase in body weight over the 8-week time course of the experiment [*F*(7,60)=557.20, *P*<.001), but no difference in total body or liver weight, body weight gain, feed efficiency ratios or food intake between diet groups (Table 1). On average (expressed as mg/day/kg body weight), the PO group consumed 244.4, 9.0 and 0, the PO+ACN group 254.0, 9.4 and 11.3; the RO group, 270.5, 1119.6 and 0; and the RO+ACN group, 264.7, 1095.8 and 11.2, of LA, ALA, and ACN respectively. There were no significant intergroup differences in LA intakes (*P*>.05).

Although a significantly higher level of serum ALA following RO consumption was observed [*F*(1,32)=4.56, *P*=.041] (Table 2a), no significant differences were found in the liver between groups [*F*(1,34)=0.02, *P*=.89] (Table 2b). EPA (% of total fatty acids) levels were approximately six-fold and eight-fold higher in serum and liver following RO (RO and RO+ACN) compared to the PO groups (PO and PO+ACN) feeding [*F*(1,32)=129.02, *P*<.001, and *F*(1,34)=70.32, *P*<.001, respectively], with two-fold higher DHA levels observed for both serum and liver [*F*(1,32)=125.30, *P*<.001, and *F*(1,34)=7.03, *P*=.012, respectively]. These higher EPA, DHA and ALA levels were reflected in the total n-3 PUFA content (Table 2a and b). A five-fold increase in DPA, an intermediary between EPA and DHA, was observed in the serum following RO (RO and RO+ACN) treatment when compared to the PO groups (PO and PO+ACN) (*P*<.001). However, such modulation was not reflected in the liver where DPA levels dropped by 50% following RO (RO and RO+ACN) treatments (*P*=.001). Conversely, serum and liver myristic acid (C14:0) and oleic acid (C18:1, n-9) levels were 20%–40% lower in the RO versus PO group (*P*<.05, Table 2a and b). No significant impact of the inclusion of ACN in either the PO or RO diets on serum or liver fatty acid status was observed (Table 2a and b).

**Fig. 2:**
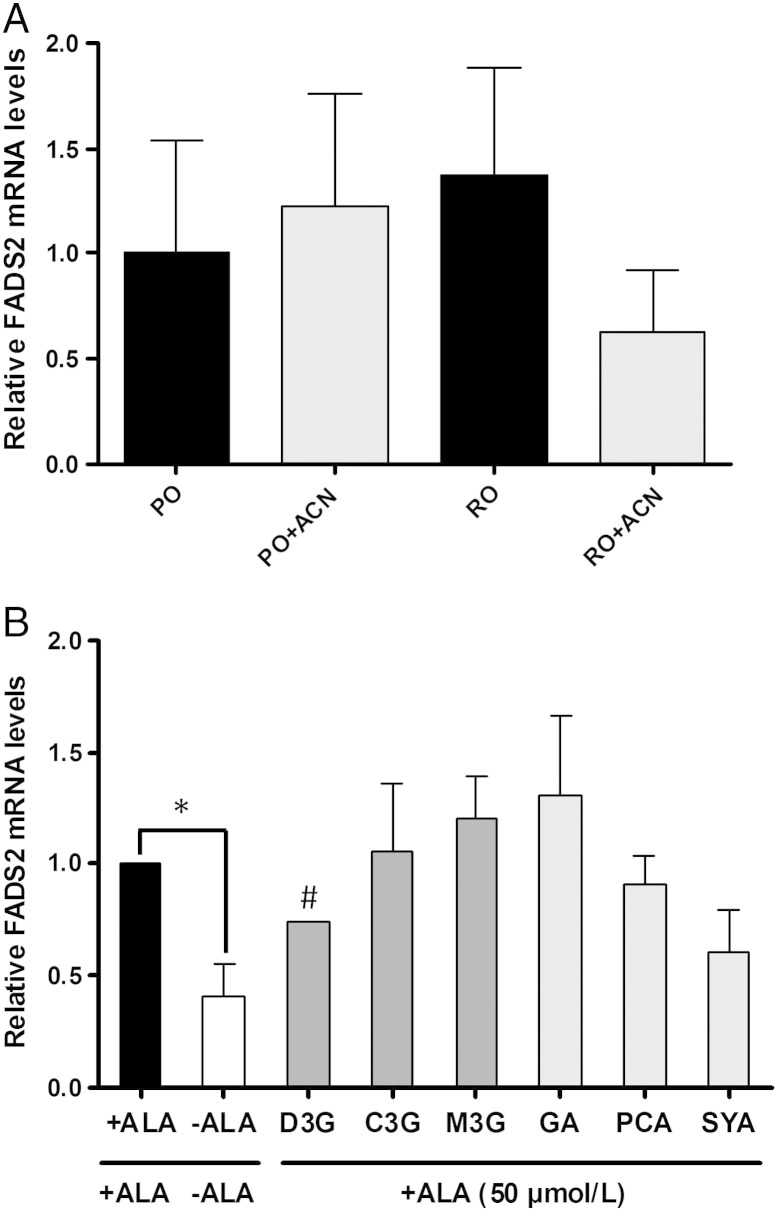
(A) mRNA expression of FADS2 in the liver. Real-time quantitative PCR analysis of mRNA expression of FADS2 in the liver of rats (*n*=8 per group) fed with PO or RO with or without ACNs (PO+ACN and RO+ACN). (B) mRNA expression of FADS2 in HepG2 cells. Real-time quantitative PCR analysis of mRNA expression of FADS2 in HepG2 cells following the addition of ALA, with or without anthocyanins or their breakdown metabolites (*n*=3). Ctrl-BSA, control using BSA; Ctrl ALA, control using ALA (50 μM); C3G, cyanindin-3-glucoside (5 μM); M3G, malvidin-3-glucoside (5 μM); D3G, dephinidin-3-glucoside (5 μM); GA, gallic acid (5 μM); PCA, p-coumaric acid (5 μM); SYA, syringic acid (5 μM). One-way ANOVA was used followed by Tukey's multiple comparison test. *Mean values were significantly different compared with control-BSA (−ALA; *P*<.05); ^#^mean values were significantly different compared with control+ALA (*P*<.05).

No significant impact of the 8-week feeding PO and RO with or without ACN on *FADS2* gene expression in liver samples was evident [*F*(3,20)=0.57, *P*=.64] (Fig. 2A).

### 3.2 Human study

Mean plasma ALA, EPA and DHA were 0.54%±0.17%, 1.15%±0.60% and 2.52%±0.62% respectively with no significant differences in the fatty acid profiles between the control and elderberry groups at baseline (Table 3). We observed a significant increase in ALA following a 12-week intervention in both the control and ACNs groups [*F*(1,23)=6.99, *P*=.015]. No significant impact of the elderberry intervention on EPA, DPA or DHA status was evident (Table 3).

### 3.3 Cell study

None of the ACNs (C3G, M3G and D3G) or their breakdown products [GA, PCA and SYA] demonstrated any cytotoxicity toward HepG2 cells at the highest concentration of 50 μM used in these experiments (data not shown). A significant increase in both ALA and EPA levels (*P*<.001) was evident following the addition of complexed BSA-ALA 50 μM in the cell culture medium. These data were supported by a two-fold increase (*P*=.014) in *FADS2* mRNA levels following ALA addition (Fig. 2B). No significant effect was observed for any of the treatments on DHA levels.

Pretreating HepG2 cells with D3G (5 μM) significantly decreased EPA levels by 43% and 61% following 24- or 48-h incubation, respectively (*P*<.0001) (Table 4). In agreement with this observation, a significant decrease in both DPA levels and *FADS2* mRNA level was also observed following treatment with D3G (Table 4 and Fig. 2B). Conversely, an increase in palmitic acid (C16:0, *P*<.001) and stearic acid (C18:0, *P*<.001) levels was observed following 48-h treatment with D3G. No significant effects were observed on fatty acid levels or *FADS2* mRNA levels in HepG2 cells challenged with any other ACNs or their breakdown metabolites (Table 4 and Fig. 2B).

## 4 Discussion

Recent prospective data suggest that the decreased risk of cardiovascular disease associated with increased fruits and vegetables intake [34–36] may be in large part attributable to intake of specific flavonoids [36]. In particular, ACNs have been reported to decrease hypertension [37] and to reduce the risk of myocardial infarction [38]. In observational studies, red wine (rich in ACNs) consumption has been associated with higher plasma and tissue EPA and DHA levels [10–13], with the only two available relevant rodent studies reporting inconsistent findings [14,15]. One of the main limitations of the two rodent studies [14,15] is the lack of purity of the tested compounds, with ACNs-rich food containing not only ACNs but also other flavonoid subclasses and phenolic acids. The present study was therefore designed to determine the biological effects of purified ACNs and their major degradation products, on both the quantity and the quality of cell membranes, plasma and liver fatty acids in a series of experiments carried out in cells and rodents. In addition, stored samples were used to examine for the first time the impact of ACNs-rich food (elderberry extract, 500 mg/day ACNs as cyanidin glycosides) on fatty acid status in a human intervention trial.

The daily estimated intake of ACNs is high (180 and 215 *mg*/day) compared with the intake of other dietary flavonoids [39–41]. For a human of 70-kg body weight, such intake corresponds to a daily consumption of 2.6 to 3.1 mg per kg body weight, respectively. During the course of our experiments in our rodent model, animals in the ACN group ingested ~11 mg/kg/day. This ACN dose was equivalent to 2.6 mg/kg/day in humans (181±22 mg ACNs in a 70-kg person) (respectively according to the human equivalent dose formula: HED=animal dose in mg/kg×(animal weight in kg/human weight in kg)^0.33^ [42,43]. Thus, the dietary intake of ACNs in our animal study was reflective of dietary achievable ACN intakes in humans. However, while ACNs intake was previously associated with 20%–35% and 10%–20% increased plasma EPA and DHA levels, respectively [14], at similar dose, we did not observe any serum or liver changes in the n-3 PUFA profile following ACNs supplementation. One possible reason for this apparent inconsistency may be attributable to the distribution profile of the ACNs. Indeed, while the major ACNs detected in the Toufektsian study were mainly glucoside derivatives of cyanidin and pelargonidin [44], our diet contained a more diverse range of ACNs i.e. glucoside derivatives of delphinidin, cyanidin, petunidin, peonidin and malvidin [44]. In addition to ACNs, the authors also reported the presence of phenylpropanoids or flavonols [45], two other subclasses of polyphenols, that may be partly responsible for the results observed both in red wine drinkers [10] and animals [14].

Traditionally, ACNs were thought to have a very low bioavailability, with <1% of the ingested amount reaching the plasma; however, some recent studies reveal that the bioavailability of these compounds may be underestimated. In particular, although intact glucosides have been detected in several mammals following oral consumption [46,47], recent evidence, however, suggests that the bioactivity of dietary ACNs is rather likely to be mediated by their degradation products [27,48,49]. In order to test this hypothesis and in our effort to screen dietary phytochemicals for potential physiological effects, we chose the pure ACNs cyanidin-3-glucoside (C3G), malvidin-3-glucoside (M3G), dephinidin-3-glucoside (D3G) and some of their major gut microbiota degradation products (i.e., GA, PCA and SYA) [25]. In our hands, C3G, M3G, or their degradation products GA, PCA or SYA did not substantially affect the distribution of fatty acid in HepG2 cells. However, supplementation of the culture medium with D3G had a reducing effect on the proportion of EPA following 48-h incubation (−60%, *P*<.0001), further substantiated by the concomitant inhibition of the Δ6-desaturase mRNA levels in our cellular model (−26%, *P*<.001). Delphinidin-rich foods, such as blackcurrant, have been previously reported to significantly reduce LC-PUFA in rodent liver tissues [50]. Since our rodent diet contains significant amounts of delphinidin (~38%), it may be hypothesized it could mask the overall impact of ACNs on DHA and EPA biosynthesis by modulating the levels of the Δ6-desaturase. Further experiments aiming at determining the exact activity of this particular compound on LC-PUFA metabolism would however be necessary before drawing solid conclusions.

In our experiments, as expected, rats fed an ACN-free diet plus RO (rich in ALA) had higher plasma and liver EPA and DHA levels than those fed with PO. Similarly, the addition of ALA 50 μM also increased EPA levels and was paralleled with two-fold increases in FADS2 activity in HepG2 cells. These results are in agreement with previous studies, where mammalian hepatocytes have been reported to accumulate high levels of EPA in cell phospholipids within 1–2 days following ALA supplementation [23,51]. However, both on our rodent or cellular models, none of the ACNs tested had any modulatory effects on LC n-3 PUFA acid biosynthesis. A possible confounding effect could be the high background ALA content in the diets of the rodents and in the cell media may have potentially ‘masked' any up-regulation of EPA and DHA synthesis associated with ACNs. However, while a more significant EPA level was previously associated with red wine consumption in the low ALA diet group [12], results derived from our rodent study showed no modulation of LC-n-3 PUFAs in the PO+ACN diet group (low ALA diet group [12]). Another possible consideration in our rodent and human trial is that our animals and participants were males and postmenopausal females, respectively, groups who are known to have substantially lower endogenous EPA and DHA biosynthesis relative to females of reproductive age [52]. Premenopausal females have a high evolutionary need for DHA and an adequate overall LC n-3 in order to meet the needs of potential pregnancies. Establishment of the impact of ACN (or other flavonoids) on LC n-3 PUFA status in premenopausal females would therefore be of interest.

In summary, our results indicate that ACNs intake is unlikely to be a meaningful determinant on LC n-3 PUFA status in mammals and suggest that their increasingly described positive impacts on a range of health outcomes is unlikely to be related to altered fatty acid metabolism. The current large deficit between worldwide fish oil production and the supplies needed to meet recommended of EPA and DHA intakes in humans merits a sustained research focus in this area, including the investigation of alternative dietary components which may increase EPA and DHA biosynthesis, bioavailability and tissue retention in fish and mammals, and the use of GM vegetable oil sources as a non-marine source of EPA/DHA.

## Authors' contribution

D.V. and A.M.M. designed the study. V.B. and B.J. conducted the animal study. N.T.H., C.O.N., V.B. and N.R. extracted and analyzed the fatty acid distribution in the rodent and human samples. J.M.S. and S.P.T. run the HepG2 cell fatty analysis. I.W. and G.R. measured desaturase gene expression in HepG2 cells. P.C. and A.C. designed and conducted the human intervention trial. D.V. and A.M.M. wrote the paper. All authors read and approved the final manuscript.

## Authors' disclosure

The authors have no conflicts of interest to disclose.

The following are the supplementary data related to this article.

Supplemental Table 1 Nutrient Composition of the experimental diets.

Supplemental Table 2 Fatty acid composition of the experimental diets.

Supplementary data to this article can be found online at http://dx.doi.org/10.1016/j.jnutbio.2014.09.005.

## Figures and Tables

**Table 1:** Growth parameters and liver weight.a,b

Growth parameters	PO	PO+ACN	RO	RO+ACN
Initial body weight (g)	376.4±28.9	369.8±22.5	355.3±27.9	355.3±19.3
Final body weight (g)	601.0±55.3	601.6±46.8	556.3±76.5	568.5±49.7
Body weight gain (g)	224.6±31.3	231.8±34.2	201.0±56.4	213.2±32.4
Food intake (g/day)	27.2±2.1	28.3±1.7	26.4±1.8	26.4±1.1
*ACN* intake (mg/day/kg)	0	11.3±1.7	0	11.2±1.2
Feed efficacy ratio	0.15±0.0	0.15±0.0	0.14± 0.0	0.14±0.0
Dietary energy intake (kJ/day)	438±34	455±28	426±30	424±18
Organ weight (g/100 g BW)				
Liver	3.18±0.3	3.29±0.4	3.30±0.6	3.10±0.2

a Diets were rich in either rich in PO or RO, not supplemented (diets PO and RO) or supplemented with 240 mg/kg ACNs (diets PO+ACN and RO+ACN).

b Values are means±S.D.; *n*=10.

**Table 2:** Main fatty acids (≥0.5%) from total lipids (weight %) of serum (a) and liver (b) of animals after 8 weeks of feeding a diet containing PO, PO+ACN, RO or RO+ACN, respectively.

Fatty acids	PO		RO		*P* value^a^		
	PO	PO+ACN	RO	RO+ACN	Lipid	ACN	L×ACN
(a) Serum							
14:0	0.8±0.2	0.8±0.2	0.6±0.1	0.6±0.2	0.002	NS	NS
16:0	25.7±4.1	25.3±4.9	22.7±2.1	23.0±2.7	0.037	NS	NS
16:1 n7	4.3±2.2	4.0±1.1	3.8±1.5	4.5±2.2	NS	NS	NS
18:0	12.1±4.0	12.5±2.4	11.7±1.4	11.3±1.1	NS	NS	NS
18:1^b^	21.9±5.9	21.2±3.6	16.5±2.4	17.5±2.9	0.003	NS	NS
18:2 n6	9.8±4.5	10.4±1.7	16.0±5.4	14.6±4.1	0.001	NS	NS
18:3 n3	1.6±1.1	1.4±1.1	2.8±1.7	2.2±1.1	0.041	NS	NS
20:4 n6	15.3±3.0	16.4±4.0	12.0±2.2	11.9±3.5	0.002	NS	NS
20:5 n3	0.4±0.2	0.5±0.1	2.7±0.8	2.5±0.7	0.000	NS	NS
22:5 n3	0.2±0.1	0.2±0.1	1.0±0.1	1.0±0.1	0.000	NS	NS
24:1	0.4±0.3	0.3±0.1	1.1±0.5	1.0±0.4	0.000	NS	NS
22:6 n3	2.4±0.7	2.1±0.5	5.0±0.8	5.6±1.2	0.000	NS	NS
Total n-3 PUFA	4.7±2.0	4.6±1.4	10.8±2.5	11.2±3.0	0.000	NS	NS
Total n-6 PUFA	27.6±6.7	29.2±5.1	30.3±6.4	28.5±6.3	NS	NS	NS
n-3/n-6 PUFA ratio	0.2±0.1	0.2±0.1	0.4±0.2	0.4±0.2	0.000	NS	NS
(b) Liver							
14:0	0.9±0.3	0.9±0.2	0.8±0.3	0.6±0.2	0.008	NS	NS
16:0	29.4±5.2	28.7±3.0	26.2±2.0	25.5±2.7	0.012	NS	NS
16:1 n7	6.3±2.7	6.1±2.2	5.2±2.0	4.5±1.3	NS	NS	NS
18:0	11.8±3.6	11.8±4.1	15.0±2.8	15.4±2.9	0.004	NS	NS
18:1^b^	28.8±6.7	30.0±7.1	20.3±4.8	20.2±3.7	0.000	NS	NS
18:2 n6	5.8±3.3	5.7±2.0	10.2±4.3	9.7±2.8	0.000	NS	NS
18:3 n3	1.2±0.8	0.9±0.5	1.1±0.5	1.5±0.5	NS	NS	NS
20:4 n6	8.9±3.9	9.6±3.5	8.0±2.4	8.2±1.9	NS	NS	NS
20:5 n3	0.2±0.2	0.2±0.1	1.7±0.7	1.6±0.6	0.000	NS	NS
22:5 n3	0.2±0.1	0.2±0.1	0.1±0.0	0.1±0.1	0.012	NS	NS
24:1	0.3±0.2	0.3±0.2	1.1±0.4	1.1±0.2	0.000	NS	NS
22:6 n3	3.3±1.7	3.2±1.2	7.2±2.2	7.7±2.6	0.000	NS	NS
Total n-3 PUFA	5.0±2.8	4.7±1.6	10.2±2.5	11.2±3.7	0.000	NS	NS
Total n-6 PUFA	16.4±7.2	17.0±5.7	20.1±6.1	20.2±4.4	NS	NS	NS
n-3/n-6 PUFA ratio	0.29±0.1	0.3±0.1	0.58±0.3	0.60±0.3	0.001	NS	NS

Results represent means±S.D. (*n*=10). Totals include some minor components not shown.

a Two-way ANOVA: lipid, significant influence of lipid source included in the treatment; ACN, significant influence of ACNs; L×ACN, interaction; NS, not significant (*P*>.05).

b Contains n-9 and n-7 isomers.

**Table 3:** Main fatty acids (≥0.5%) from total lipids (weight %) of plasma from a human randomized controlled trial where participants consumed an elderberry extract or a placebo for 12 weeks.

Fatty acid	Control		Elderberry		*P* value^a^		
	Week 0	Week 12	Week 0	Week 12	Time	ACN	T×ACN
14:0	10.62±4.5	9.05±4.8	8.76±3.0	8.45±4.7	NS	NS	NS
16:0	18.82±1.6	18.58±1.5	19.53±1.6	20.33±1.5	NS	0.014	NS
16:1^b^	2.01±0.5	2.25±0.6	2.43±0.6	2.76±0.7	NS	0.010	NS
18:0	6.95±0.6	6.97±0.5	6.77±0.8	7.41±1.0	NS	NS	NS
18:1^b^	17.96±2.0	18.00±2.2	19.14±1.7	18.96±2.6	NS	NS	NS
18:2n6	24.64±4.0	25.90±3.2	24.52±3.8	22.48±3.5	NS	NS	NS
18:3n3	0.49±0.2	0.67±0.2	0.59±0.2	0.65±0.2	0.015	NS	NS
20:3n6	1.42±0.5	1.31±0.3	1.34±0.6	1.34±0.3	NS	NS	NS
20:4n6	5.38±1.7	5.53±1.4	5.91±1.8	5.28±1.3	NS	NS	NS
20:5n3	1.29±0.7	1.31±1.0	0.99±0.5	1.31±1.1	NS	NS	NS
22:5n3	0.60±0.1	0.58±0.1	0.58±0.2	0.58±0.2	NS	NS	NS
22:6n3	2.67±0.7	2.51±0.9	2.37±0.6	2.49±1.0	NS	NS	NS
Total n-3 PUFA	1.26±0.3	1.27±0.5	1.13±0.3	1.26±0.5	NS	NS	NS
Total n-6 PUFA	4.61±0.7	4.84±0.6	4.67±0.5	4.39±0.6	NS	NS	NS
n-3/n-6 PUFA ratio	0.28±0.1	0.26±0.1	0.24±0.1	0.29±0.1	NS	NS	NS
Δ6 (18:3 n-6/18:2 n-6)	0.02±0.0	0.02±0.0	0.02±0.0	0.03±0.0	0.017	NS	NS

Values are means±S.D. (*n*=13). Totals include some minor components not shown.

a Two-way ANOVA: time, significant influence of duration of the trial; ACN, significant influence of ACNs; T×ACN, interaction; NS, not significant (*P*>.05).

b Contains n-9 and n-7 isomers.

**Table 4:** Effects of ALA, ACNs and their metabolites on the fatty acid composition of HepG2 cell membranes after 24- or 48-h incubation.^a^

Fatty acid	Ctrl-BSA		Ctrl ALA		C3G		M3G		D3G	
	24 h	48 h	24 h	48 h	24 h	48 h	24 h	48 h	24 h	48 h
16:0	28.2±0.2	26.4±0.2	27.2±0.1	27.5±0.4	29.5±2.5	26.7±1.2	27.4±0.7	30.1±2.9	27.5±0.1	31.2±4.1^b^
16:1	4.9±0.7	4.4±0.8	3.0±0.0^c^	2.6±0.2^c^	2.1±0.4	2.6±0.2	3.0±0.1	2.3±0.4	2.7±0.0	1.5±0.8^b^
18:0	22.3±1.7	22.0±1.3	23.2±1.3	24.2±1.3	29.7±8.5	23.0±1.0	22.9±1.7	29.8±7.4	22.5±2.0	32.7±10^b^
18:1	36.7±1.5	39.8±1.1	28.2±0.2^c^	29.9±0.2^c^	23.1±6.0	31.2±2.0	29.9±1.1	23.7±6.9	27.8±0.2	20.5±10.3^b^
18:2n6	3.1±0.5	3.0±0.4	3.3±0.9	3.2±0.7	2.5±0.0	3.5±1.2	3.2±0.7	2.6±0.4	3.4±0.7	2.0±0.4
18:3n3	0.2±0.2	0.1±0.0	7.9±1.8^c^	5.2±0.6^c^	7.1±3.5	5.9±1.5	6.6±2.6	4.9±1.3	9.1±2.4	4.7±2.8
20:0	0.3±0.0	0.3±0.1	0.3±0.1	0.3±0.1	0.4±0.1	0.3±0.1	0.3±0.1	0.3±0.1	0.3±0.1	0.3±0.1
20:1	0.8±0.1	0.9±0.2	0.7±0.1	0.7±0.1^c^	0.5±0.0	0.6±0.1	0.6±0.1	0.5±0.1	0.6±0.1	0.4±0.1^b^
20:4n6	1.6±0.1	1.6±0.1	2.8±0.1	2.8±0.1	2.5±0.7	3.1±0.4	2.8±0.0	2.2±0.6	3.3±0.2	2.4±1.2
20:5n3	0.3±0.0	0.2±0.0	1.9±0.6^c^	2.3±1.0^c^	1.1±0.5	1.6±0.7	1.9±0.5	2.0±1.3	1.1±0.3	0.9±0.6^b^
24:1	0.2±0.0	0.3±0.0	0.3±0.0	0.3±0.1	0.3±0.1	0.3±0.0	0.3±0.1	0.3±0.1	0.2±0.1	0.3±0.0
22:6n3	1.0±0.0	0.8±0.0	0.9±0.1	0.8±0.1	0.9±0.2	0.8±0.1	0.8±0.0	0.9±0.1	1.0±0.1	3.0±2.2
n-3 PUFA	1.5±0.2	1.1±0.0	10.7±2.5^c^	8.3±1.7^c^	9.2±4.2	8.3±1.8	9.3±3.2	7.8±2.7	11.2±2.6	8.6±1.2
n-6 PUFA	4.7±0.6	4.6±0.6	6.1±0.9	5.9±0.9	5.0±0.7	6.6±1.1	6.0±0.7	4.8±0.8	6.8±0.5	4.3±1.5^b^
n-3/n-6 ratio	0.3±0.1	0.2±0.0	1.8±0.7^c^	1.5±0.5^c^	1.8±0.6	1.3±0.4	1.6±0.7	1.6±0.4	1.7±0.5	2.2±0.5


Fatty acid	GA		PCA		SYA		*P* value^d^		
	24 h	48 h	24 h	48 h	24 h	48 h	Time	Treatment	T×T
16:0	27.0±0.2	29.7±2.4	27.3±0.2	26.3±0.5	27.4±0.1	26.9±0.7	NS	NS	0.004
16:1	3.0±0.0	2.0±0.6	3.0±0.1	2.8±0.1	3.2±0.1	2.9±0.1	0.000	0.000	0.030
18:0	21.5±1.8	30.9±7.5	21.2±1.5	22.4±0.8	20.8±0.9	22.9±0.7	0.03	NS	0.020
18:1	29.2±0.3	23.6±6.2	29.8±0.2	32.0±0.3	30.5±0.8	31.6±0.6	NS	0.000	0.009
18:2n6	3.5±0.8	2.5±0.1	3.5±0.9	3.3±0.8	3.5±0.8	3.2±0.7	NS	NS	NS
18:3n3	8.3±1.6	5.0±1.6	8.3±1.9	5.4±0.6	7.7±1.7	5.0±0.9	0.000	0.000	NS
20:0	0.3±0.1	0.3±0.1	0.3±0.1	0.3±0.1	0.3±0.1	0.3±0.1	NS	NS	NS
20:1	0.7±0.1	0.4±0.0	0.6±0.1	0.7±0.1	0.4±0.2^b^	0.7±0.1	NS	0.000	0.003
20:4n6	3.3±0.2	2.5±0.3	3.0±0.0	3.0±0.2	2.9±0.0	2.7±0.2	0.04	0.000	0.020
20:5n3	1.6±0.5	1.8±1.1	1.6±0.5	2.2±0.9	1.9±0.7	2.3±1.0	NS	0.001	NS
24:1	0.2±0.0	0.2±0.0	0.3±0.0	0.3±0.0	0.2±0.0	0.3±0.0	NS	NS	NS
22:6n3	0.9±0.1	0.6±0.2	0.8±0.0	0.8±0.0	0.8±0.0	0.8±0.0	0.000	NS	0.040
n-3 PUFA	10.8±2.0	7.4±2.9	10.7±2.4	8.4±1.6	10.4±2.3	8.1±1.9	0.006	0.000	NS
n-6 PUFA	6.8±0.5	5.0±0.1	6.5±0.9	6.3±1.0	6.4±0.9	5.9±1.0	0.020	0.030	0.003
n-3/n-6 ratio	1.6±0.4	1.5±0.5	1.7±0.6	1.4±0.5	1.7±0.6	1.5±0.6	NS	0.000	NS

Ctrl-BSA, control using BSA; Ctrl ALA, control using ALA (50 μM); C3G, cyaniding-3-glucoside (5 μM); M3G, malvidin-3-glucoside (5 μM); D3G, dephinidin-3-glucoside (5 μM); GA (5 μM); PCA, p-coumaric acid (5 μM); SYA, syringic acid (5 μM).

a Values are means±S.D. (*n*=3).

b Mean values were significantly different compared with control-ALA (*P*<.05).

c Mean values were significantly different compared with control-BSA (*P*<.05).

d Two-way ANOVA: time, significant influence of incubation time (24 h or 48 h) on fatty acid composition; Treatment, significant influence of BSA, ALA, ACNs or their metabolites on fatty acid composition; T×T, interaction. NS, not significant (*P*>.05).
