# Supplementary material for: Anthocyanins do not influence long-chain n-3 fatty acid status: studies in cells, rodents and humans
Source: J Nutr Biochem. 2015 Mar;26(3):211–8. doi: 10.1016/j.jnutbio.2014.09.005 (PMC4336242; doi:10.1016/j.jnutbio.2014.09.005)
Supplement: Supplemental Table 1 — Nutrient Composition of the experimental diets. [file mmc1.docx]

Supplemental Table 1: Nutrient Composition of the experimental diets

| Component (g/kg diet) | Amount | |
| --- | --- | --- |
|  | **PO^1^** | **RO^1^** |
|  |  |  |
| Casein | 190 | 190 |
| Corn starch | 480 | 480 |
| Sucrose | 65 | 65 |
| Cellulose | 47 | 47 |
| Mineral mix^2^ | 9 | 9 |
| Calcium^3^ | 17.5 | 17.5 |
| Choline bitartrate | 1.9 | 1.9 |
| Vitamin mix^4^ | 9 | 9 |
| Rapeseed oil | 0 | 42 |
| Palm oil | 42 | 0 |

^1^ Diet PO, palm oil; Diet RO, rapeseed oil

^2^ Mineral mix S10026 supplied in g/kg of premix triturated in sucrose: NaCl, 259; MgO, 41.9; MgSO_4_·7H_2_O, 257.6; (NH_4_)2MoO_4_·4H_2_O, 0.3; CrK(SO_4_)_2_ · 12H_2_O, 1.925; CuCO_3_, 1.05; C_6_H_5_FeO_7_, 21; MnCO_3_ · xH2O, 12.25; KI, 0.035; NaF, 0.2; Na_2_SeO_3_, 0.035; [ZnCO_3_]_2_, 5.6.

^3^ Combination of dicalcium phosphate (CaHPO_4_) and calcium carbonate (CaCO_3_)

^4^ AIN-76A vitamin mix supplied in g/kg of premix triturated in sucrose: vitamin A acetate (500,000 IU/gm), 0.8; vitamin D3 (100,000 IU/gm), 1; vitamin E acetate (500 IU/gm), 10; menadione sodium bisulfite, 0.08; biotin (1%), 2; cyanocobalamin (0.1%), 1; folic acid, 0.2; nicotininc acid, 3; calcium pantothenate, 1.5; pyridoxine-HCl, 0.7; riboflavin, 0.6; thiamine-HCl, 0.6.
