# Supplementary material for: Anthocyanins do not influence long-chain n-3 fatty acid status: studies in cells, rodents and humans
Source: J Nutr Biochem. 2015 Mar;26(3):211–8. doi: 10.1016/j.jnutbio.2014.09.005 (PMC4336242; doi:10.1016/j.jnutbio.2014.09.005)
Supplement: Supplemental Table 2 — Fatty acid composition of the experimental diets. [file mmc2.docx]

Supplemental Table 2: Fatty acid composition of the experimental diets

| Fatty acid | Common name | PO^1^ | RO^1^ |
| --- | --- | --- | --- |
| C12:0 | Lauric | 10 | 0 |
| C14:0 | Myristic | 30 | 0 |
| C16:0 | Palmitic | 1610 | 260 |
| C18:0 | Stearic | 220 | 150 |
| C20:0 | Arachidic | 20 | 0 |
| C16:1(n-7) | Palmitoleic | 0 | 0 |
| C18:1(n-9) | Oleic | 1790 | 730 |
| C18:2(n-6) | Linoleic | 540 | 570 |
| C18:3(n-3) | Linolenic | 20 | 2360 |
| C20:4(n-6) | Arachidonic | 0 | 0 |
| C20:5(n-3) | Eicosapentaenoic | 0 | 0 |
| C22:5(n-3) | Docosapentaenoic | 0 | 0 |
| C22:6(n-3) | Docosahexaenoic | 0 | 0 |
|  |  |  |  |
| Total SFA^2^ |  | 1790 | 410 |
| Total MUFA^2^ |  | 1800 | 730 |
| Total PUFA^2^  MUFA/SFA  PUFA/SFA |  | 560  1.0  0.3 | 2930  1.8  7.2 |
| Total (n-3) |  | 20 | 2360 |
| Total (n-6) |  | 540 | 570 |
| n-3/n-6 |  | 0.0 | 4.1 |

^1^ Supplied in mg/100 g food. Diet PO (Palm oil); Diet RO (Rapeseed oil).

^2^ Abbreviations: SFA, saturated fatty acids; MUFA, monounsaturated fatty acids; PUFA, polyunsaturated fatty acids.
